# Applications of Learning Analytics in High Schools: A Systematic Literature Review

**DOI:** 10.3389/frai.2021.737891

**Published:** 2021-09-27

**Authors:** Erverson B. G. de Sousa, Bruno Alexandre, Rafael Ferreira Mello, Taciana Pontual Falcão, Boban Vesin, Dragan Gašević

**Affiliations:** ^1^ Cesar School, Recife, Brazil; ^2^ Departamento de Computação, Universidade Federal Rural de Pernambuco, Recife, Brazil; ^3^ School of Business, Department of Business, History and Social Sciences, University of South-Eastern Norway, Vestfold, Norway; ^4^ Department of Computer Science, Faculty of Information Technology and Electrical Engineering, Norwegian University of Science and Technology, Trondheim, Norway; ^5^ Centre for Learning Analytics, Faculty of Information Technology, Monash University, Clayton, VIC, Australia; ^6^ School of Informatics, University of Edinburgh, Edinburgh, United Kingdom; ^7^ Faculty of Computing and Information Technology, King Abdulaziz University, Jeddah, Saudi Arabia

**Keywords:** learning analytics, high school, teaching and learning, learning environments, machine learning

## Abstract

Learning analytics aims to analyze data from students and learning environments to support learning at different levels. Although learning analytics is a recent field, it reached a high level of maturity, especially in its applications for higher education. However, little of the research in learning analytics targets other educational levels, such as high school. This paper reports the results of a systematic literature review (SLR) focused on the adoption of learning analytics in high schools. More specifically, the SLR followed four steps: the search, selection of relevant studies, critical assessment, and the extraction of the relevant field, which included the main goals, approaches, techniques, and challenges of adopting learning analytics in high school. The results show that, in this context, learning analytics applications are focused on small-scale initiatives rather than institutional adoption. Based on the findings of this study, in combination with the literature, this paper proposes future directions of research and development in order to scale up learning analytics applications in high schools.

## 1 Introduction

Over the last several years, technology has become an essential tool to support students and instructors in creating more effective educational experiences. In this context, the propagation of online learning environments (e.g., learning management systems, student diaries, library systems, digital repositories, and academic systems) has increased significantly, expanding the data generated about the educational process (Gaftandzhieva et al., 2020). These digital footprints can assist teaching and learning practices to foster better student achievement (Varanasi et al., 2018) and support teachers’ practices (Jivet et al., 2018).

To reach the potential of analysis of this data, learning analytics emerged as a field that focuses on collecting, analyzing, and reporting data about learners and contexts in which learning occurs (Siemens and Gasevic, 2012). The use of learning analytics can bring concrete benefits for students, teachers and institutions. The large amount of student data, such as demographic information, grades and student behaviors, expands the possibilities of retention strategies and academic success, thus moving away from leveling by the average, to meet the needs of each student in a personalized and data-oriented way (Tan et al., 2016; Aguerrebere et al., 2017).

Learning analytics has been widely researched and used in higher education institutions, especially due to the maturity level of adopting data analysis tools in these institutions (Leitner et al., 2017; Waheed et al., 2018; Charitopoulos et al., 2020). However, despite some promising results, learning analytics does not have the same level of adoption in other educational contexts, such as high schools (Cechinel et al., 2020; Ifenthaler, 2021). That is a limitation, as the adoption of educational technology in these levels of education has created environments where rich information could be extracted from the generated data (Schmid and Petko, 2019). For instance, Wastiau et al. (2013) performed a survey in Europe that demonstrated the importance of digital technologies in a middle school. Moreover, several recent papers are focused on the application of data analysis for high school students (Wastiau et al., 2013; Bernhardt, 2017; Martínez-Abad et al., 2020). While higher education institutions are quick to adopt learning analytic tools such as extensions of educational governance, teachers in high schools are often skeptical of the politics and utility of learning analytic tools, and often resist their implementation through their academic practice (Brown, 2020). Since the increased capacity of educational data mining has created a boost of educational technology tools development, Brown (2020) expressed the need to investigate how learning analytic tools shape activities beyond the classroom, and how they further influence curriculum and pedagogy.

There are many educational challenges in the high school context that involve all stakeholders in teaching and learning processes (Gaftandzhieva et al., 2020). Learning analytics can be used to address these challenges, such as school dropout (Khalil and Ebner, 2015), the difficulty of collaboration among students (Berland et al., 2015), the development of scientific argumentation and writing (Lee et al., 2019; Palermo and Wilson, 2020), and the development of computational thinking, which is an emerging ability for this age group (Grover et al., 2017). Teachers can be supported in understanding student practices and classroom variations (Quigley et al., 2017) and in monitoring students’ motivation levels (Aluja-Banet et al., 2019). Managers and decision-makers can use learning analytics to identify students who are in the vulnerable situation of not being able to graduate on time (Aguiar et al., 2015; Jiménez-Gómez et al., 2015) and in developing curricula that meet students’ needs and expectations (Monroy et al., 2013).

Based on this context and in the fact that several literature reviews present the potential in using learning analytics in different educational contexts, such as higher education, professional and workplace learning, vocational education, for massive open online courses, but not for the high school context, this paper presents a systematic literature review focusing on the applications of learning analytics 59 in high schools. The SLR enables the identification, evaluation, and interpretation of previous works that provide details about methods, tools and use of learning analytics in this context. More specifically, this review aims to provide a broad description of the main approaches, educational goals, techniques and challenges related to learning analytics and high schools.

The following sections of the paper present: section 2, a short background on learning analytics and previous literature reviews on the topic; section 3, the research questions investigated in this literature review; section 4, details about the method used; sections 5, 6, the results and the discussion of the finding in this study; finally, section 7, the limitations of the proposed literature review.

## 2 Learning Analytics

The most popular definition of learning analytics was presented by the Society for Learning Analytics Research (SoLAR) at the First Learning Analytics and Knowledge Conference in 2011—LAK′11 (Long et al., 2011). Learning analytics is defined as “the measurement, collection, analysis, and reporting of data about learners, learning environments and contexts to understand and optimize learning and their environments” (Siemens and Gasevic, 2012). Online learners leave behind data traces, and learning analytics can gather this data from different sources and learner activities, then analyze and provide meaningful insights and visualizations for institutional managers, teachers, and learners (Gedrimiene et al., 2020).

Despite the presumable advantages of using learning analytics, few publications explore the benefits of the learning analytics field in high schools (Ifenthaler, 2021). Although LA could address several challenges faced by high schools (e.g., student dropout and supporting the development of computational thinking abilities), it was not consistently used across different institutions (Charitopoulos et al., 2020; Ifenthaler, 2021). This fact could be a result of the lack of studies analyzing the context and the potential of LA for high schools, and the shortage in involving different stakeholders in the process of adoption of LA tools [as it is done in higher education (Maldonado-Mahauad et al., 2018; Tsai et al., 2018)]. Therefore, it is necessary to bridge the gap between technological capacity and tangible improvements in teaching-learning experiences. Given this context, it is very important to identify reports of experiences that allow knowing the practical consequences of the application of learning analytics (Slotta and Acosta, 2017).

Several literature reviews about learning analytics have been published in the last 11 years since the first edition of the LAK conference. For instance, Charitopoulos et al. (2020) synthesize the main methods and techniques adopted to support data analysis using learning analytics, based on papers published between 2010 and 2018. Waheed et al. (2018) presented a bibliometric analysis of the field in order to analyze publication counts, citation counts, co-authorship patterns, citation networks, and term co-occurrence. Among the main conclusions, the authors stated that higher education institution is a common keyword in the field.

Besides these general reviews, the most common topics of the previous SLR are related to specific methods, especially related to the development of visualizations and dashboards (Matcha et al., 2019b). In short, the main goal of these studies is to present current applications and tools to develop learning analytic visualizations, how students and instructors could benefit from learning analytic dashboards in practice, and the challenges and future research lines.

Previous studies also described how learning analytics developed in specific world regions. For instance, Cechinel et al. (2020) and Pontual Falcão et al. (2020) list several research initiatives and practical applications of learning analytics in Latin America. Similarly, Ferguson et al. (2015) described the perspectives of the adoption of learning analytics in Europe. All studies reported a major use in Higher Education Institutions in comparison to other levels of education.

Finally, other reviews report on the use of learning analytics in higher education (Leitner et al., 2017; Viberg et al., 2018), professional and workplace learning (Ruiz-Calleja et al., 2017; ?), and vocational education (Gedrimiene et al., 2020). However, to the best of our knowledge, there are no previous systematic literature reviews on adopting Learning Analytics at high schools, which is this paper’s main goal.

Moreover, it is important to remark that the high school context is significantly different from the educational settings of the previous reviews. For instance, the students in high school are usually under 18 years (different from higher education and professional learning), which could raise different ethical concerns and needs for LA. Besides, in general, the high school teachers’ technology backgrounds are not the same as professors at the universities. Finally, the data collected from students in high schools do not involve many interactions with learning management systems or MOOC platforms, which are the primary data collected for LA application.

## 3 Research Questions

The objective of this SLR was to identify primary studies that focus on the use of Learning Analytics techniques aiming at solving high school problems. Based on this context, this study addresses the following research questions:

**RESEARCH QUESTION 1 (RQ1):** *What are the educational goals of using learning analytics in high schools?*

The first research question focuses on the fact that the primary purpose of using Learning Analytics is educational rather than technological (Gašević et al., 2015). Therefore, this review starts by highlighting the educational motivation and problems that lead to the adoption of learning analytics in high schools. More specifically, we evaluated a subset of the categories proposed in previous works (Moissa et al., 2015; Viberg et al., 2018; Cechinel et al., 2020), such as predicting and enhancing students learning outcomes, analyzing students’ learning processes, supporting teachers’ decisions and reflection, and support writing activities. Subsequently, as the application of learning analytics in general means using data analysis, we intended to analyze the data processing approaches used in the learning analytics adoption process. Thus, our second research question was:

**RESEARCH QUESTION 2 (RQ2):** *What are the approaches for the use of learning analytics in high schools?*

To answer the second research question, we adopted the categories proposed by previous works (Viberg et al., 2018): 1) **Prediction**: the use of regression and classification techniques to predict learning outcomes; 2) **Clustering**: application of different unsupervised methods to group similar instances of the data (e.g., students or learning material); 3) **Relationship mining**: this category includes methods related to association rule mining, sequential pattern mining, process mining, and casual data mining; 4) **Distillation of data for human judgment**: methods in this category include visualizations (e.g., dashboards) and statistical analysis to assist humans to make sense of the findings and support decision making; 5) **Discovery with models**: this category describes the application of models proposed in previous study, but analyzing new data to discover more patterns.

After understanding the approach of using learning analytics, we intended to identify the leading machine learning algorithms that have been implemented in the development of learning analytics systems for high schools. This analysis is essential as machine learning approaches are largely used by the LA community (Charitopoulos et al., 2020). Therefore, we intend to investigate the leading algorithms used and if they are aligned with the algorithms proposed for other educational contexts (Leitner et al., 2017; Ruiz-Calleja et al., 2017, 2021). As such, our third research question is:

**RESEARCH QUESTION 3 (RQ3):** *Which machine learning techniques have been used to support learning analytic systems in high schools?*

In order to evaluate the potential of using LA for high school, the fourth research question focuses on describing the evidence of learning analytics research in high schools. Previous literature performed a similar evaluation for higher education success (Viberg et al., 2018). In this case, we analyzed if the selected papers offered evidence of a positive or negative impact of using learning analytics and if they presented (or not) empirical evaluation to support this evidence.

**RESEARCH QUESTION 4 (RQ4):** *What evidence, if any, shows that Learning Analytics improves the performance of students in high schools?*

Finally, we also looked into the main challenges in using learning analytics reported by the studies retrieved in this literature review. This research question aims to make research aware of potential issues with the adoption of LA to high schools and provide a direction on how to avoid them. As such, the last research question is:

**RESEARCH QUESTION 5 (RQ5):** *What are the challenges in using learning analytics in high schools?*

## 4 Methods

The SLR developed in this study followed the guidelines proposed by Kitchenham (Kitchenham, 2004). The review focused on the literature of the last 10 years, papers published between 2010 and 2020. The method adopted is composed of five steps: 1) definition of the research questions, 2) definition of the search strategies, 3) article selection process, 4) critical assessment, and 5) extraction of relevant fields. In the following sections, details about each step are presented.

### 4.1 Search Strategies

The first step in the systematic review was a keyword search. This study explored five academic databases to conduct the search: ACM Digital Library, IEEE Xplore, ScienceDirect, SpringerLink, and Scopus. The selection of these databases was based on the literature (Matcha et al., 2019b) and assuring the inclusion of the conference and journal maintained by the Society of Learning Analytics Research - SoLAR^1^ as the most prominent specialized publication avenues for research in learning analytics. The search on all included databases was performed on January 4, 2021.

The query *“learning analytics” AND “high school”* was applied to each academic database cited above to conduct the search. To obtain a wider range of papers in this initial interaction, the keywords were applied for all fields of the article, not restricted to title and abstract. Table 1 presents the number of papers retrieved per database.

**TABLE 1:** Counts of studies found in each database

Database	Studies initially retrieved	Studies selected
Scopus	1,270	30
ACM Digital Library	234	7
IEEE Xplore	6	0
ScienceDirect—Elsevier	134	3
SpringerLink	522	2
**Total**	**2,166**	**42**

### 4.2 Selection Process

The second step of the review was carried out to exclude papers out of the scope. In this review, only primary studies published in journals, conferences, or workshops about applying learning analytics to improve teaching and learning in the context of high schools were included. Moreover, studies that were not published in the English language nor available online were excluded.

**FIGURE 1:**
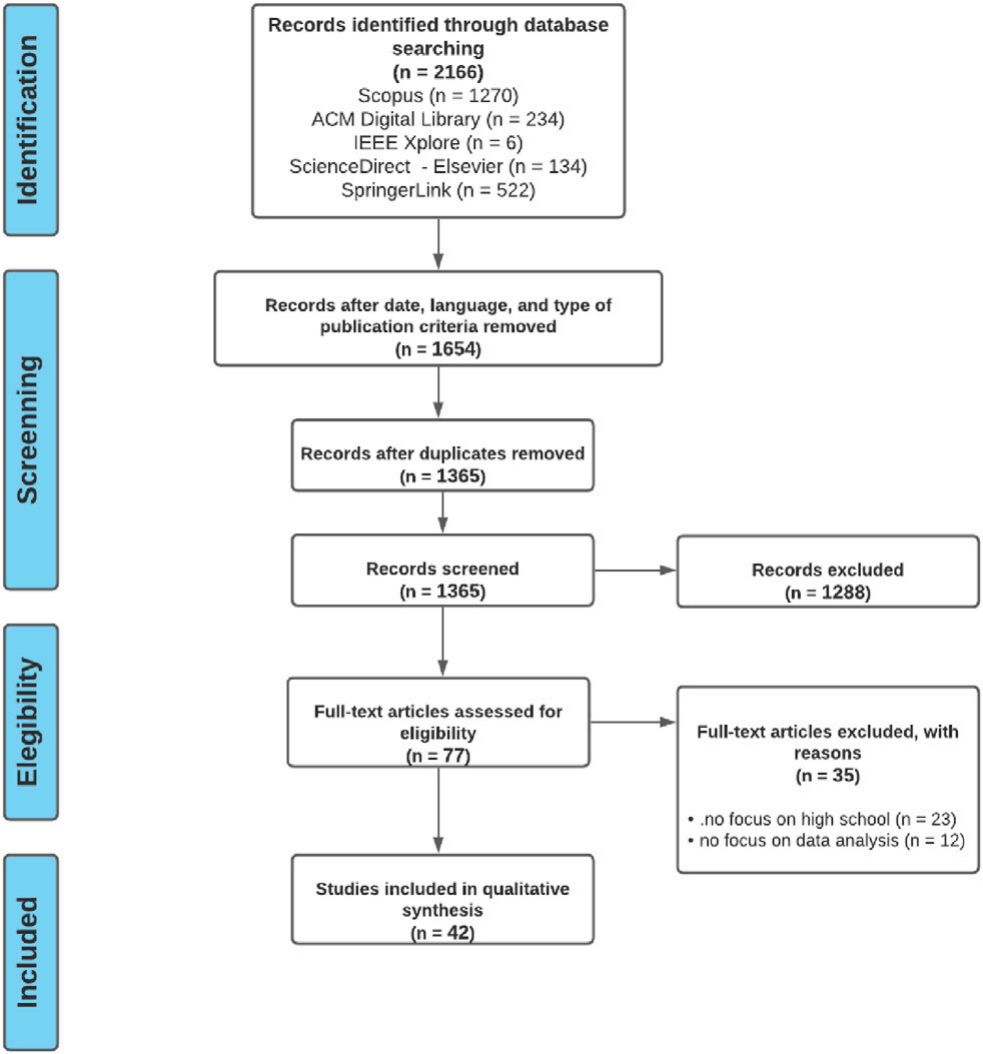
PRISMA Flowchart. The PRISMA flow diagram for the systematic review detailing the database searches, the number of abstracts screened, and the full texts retrieved.

The initial interaction removed 512 papers out of the 2,166 originally retrieved because they were not published in journals, conferences, or workshops in English between 2010 and 2020. Subsequently, the remaining 1,654 papers were imported into *Rayyan*, a free web tool designed to help researchers work on systematic reviews and dramatically accelerate the process of screening and selecting studies (Ouzzani et al., 2016). Using Rayyan, a three-step process was performed: 1) revise and remove the duplicates suggested by the tool, 2) check the pertinence of the papers to the topics of this review using title and abstract; 3) check the pertinence of the papers to the topics of the review using introduction and final considerations. At the end of this process, 42 studies (24 conference paper and 18 journal papers) were considered relevant for this SLR, as described in Table 1. Figure 1 presents a summary of each step and the number of articles selected in each phase. We calculated the agreement between the two coders based on their categorization of the papers into relevant or not for this SLR.

### 4.3 Critical Assessment

In addition to the selection process described in section 4.2, Kitchenham (2004) recommend that an SLR should also evaluate the quality of the selected papers in order to validate the results found. Kitchenham (2004) indicate three types of quality measures that should be addressed: bias, internal validity, and external validity. However, the same authors point out that depending on the topic of the SLR, the questions could cover different aspects. Therefore, this step does not eliminate any articles, but it can indicate which ones are more likely to be relevant for the discussion.

In terms of bias, this SRL focused on the analysis of the studies’ conceptualization and nature. For internal validity, the goal was to evaluate the methodology of the paper. Finally, the external validity focused on the generalizability of the proposed solution. Table 2 presents the details of each question assessed. For each paper selected, the authors evaluated the questions as a yes or no answer using 1 and 0, respectively.

**TABLE 2:** Questions used to evaluate the quality of the selected studies.

ID	Type	Question
Q1	Bias	Did the study present a research project and not an expert opinion?
Q2	Bias	Did the study fully describe the context analyzed?
Q3	Internal Validity	Were the objectives of the study clearly defined?
Q4	Internal Validity	Was the proposed methodology adequate to achieve the research objectives?
Q5	Internal Validity	Were the data collection methods used and described correctly?
Q6	External Validity	Have the research results been properly validated?
Q7	External Validity	Did the study explain and discuss how Learning Analytics could improve the teaching and learning process in high schools?

Table 3 presents the results of the quality criteria evaluation. Each row represents an article, and the columns “Q1′” to “Q7” represent the seven criteria defined by the questions presented in section 4.3.

**TABLE 3:** Critical assessment results of the primary studies.

Study	Q1	Q2	Q3	Q4	Q5	Q6	Q7	Total
Berland et al. (2015)	1	1	1	1	1	1	1	7
Lee et al. (2019)	1	1	1	1	1	1	1	7
Chen, (2020)	1	1	1	1	1	1	1	7
Jiménez-Gómez et al. (2015)	1	1	1	1	1	1	1	7
d’Anjou et al. (2019)	1	1	1	1	1	1	1	7
Admiraal et al. (2020)	1	1	1	1	1	1	1	7
Papamitsiou and Economides, (2015)	1	1	1	1	1	1	1	7
Ruipérez-Valiente and Kim, (2020)	1	1	1	1	1	0	1	6
Tan et al. (2016)	1	1	1	1	1	0	1	6
Palermo and Wilson, (2020)	1	1	1	1	1	0	1	6
Wen et al. (2018)	1	1	1	1	1	0	1	6
Grover et al. (2017)	1	1	1	0	1	1	1	6
Quigley et al. (2017)	1	1	1	1	0	1	1	6
Blasi, (2017)	1	0	1	1	1	1	1	6
Marcu and Danubianu, (2020)	1	1	0	1	1	1	1	6
Manske and Hoppe, (2016)	1	1	0	1	1	1	1	6
Ponciano et al. (2020)	1	1	0	1	1	1	1	6
Allen et al. (2017)	1	1	0	1	1	1	1	6
Aguiar et al. (2015)	1	1	0	1	1	1	1	6
Lakkaraju et al. (2015)	1	1	1	1	1	1	0	6
Yousafzai et al. (2020)	1	1	1	1	1	1	0	6
Tomkins et al. (2016)	1	1	1	1	1	1	0	6
Aluja-Banet et al. (2019)	1	1	1	1	0	0	1	5
Slotta and Acosta, (2017)	1	1	1	1	0	0	1	5
Seitlinger et al. (2020)	1	1	1	0	0	1	1	5
Monroy et al. (2013)	1	1	0	1	1	0	1	5
Khalil and Ebner, (2015)	1	1	0	0	1	1	1	5
Mayfield and Butler, (2018)	1	1	0	0	1	1	1	5
Aguerrebere et al. (2017)	1	1	0	0	1	1	1	5
Xie et al. (2014)	1	1	0	0	1	1	1	5
Sindhgatta et al. (2017)	1	1	0	1	0	1	1	5
Ostrow et al. (2016)	1	0	0	1	1	1	1	5
Coleman et al. (2019)	1	1	1	1	1	0	0	5
Filho and Adeodato, (2019)	1	1	1	1	1	0	0	5
Ashenafi et al. (2016)	1	1	0	1	1	1	0	5
Uzel et al. (2018)	1	1	0	1	1	1	0	5
Martin et al. (2013)	1	1	0	0	1	1	1	5
Gaftandzhieva et al. (2020)	1	1	1	0	0	0	1	4
Wandera et al. (2019)	1	1	0	1	1	0	0	4
Jadav et al. (2017)	1	1	0	0	1	1	0	4
Baker et al. (2020)	1	1	0	0	1	1	0	4
Lailiyah et al. (2019)	1	1	0	0	0	0	1	3

Most studies examined how learning analytics could improve teaching and learning in high schools and answer the research questions in this review. Only two studies [(Blasi, 2017) and (Ostrow et al., 2016)] did not adequately present the study context. Nineteen papers did not clearly define their study objectives. A small part, seven of 42 studies, did not clearly describe their data collection methods. Seven studies obtained the maximum score, and the vast majority of studies obtained grades 6 and 5 out of 7. The highest number of negative responses was found for (Lailiyah et al., 2019).

### 4.4 Extraction of Relevant Fields

Finally, the last step of the SLR was the extraction of the relevant information from the full text of the selected articles. To do so, both coders read the full text of the papers to collaboratively extract the information. The information analyzed encompasses the answers to the research questions and demographic data about the paper. Table 4 shows all the fields that were extracted from the articles.

**TABLE 4:** Information extracted from the papers included in the systematic literature review*.*

#	Field	Description
1	ID	Unique identifier for the study
2	Title	Title of the paper
3	Authors	Authors of the paper
4	Year	Year of publication
5	Country	Country of the first author of the paper
6	Type	Conference, journal and workshop paper
7	Keywords	The keywords listed by the authors in the paper
8	Main results	Main results presented in the paper
9	Information about the quality analysis	Criteria presented in section 4.3
10	RQ1	Educational goals
11	RQ2	Learning analytics approaches applied in high schools
12	RQ3	Machine learning techniques applied in high schools
13	RQ4	Evidence that LA could improve student performance
14	RQ5	Main challenges for learning analytics in high schools

## 5 Results

### 5.1 Quantitative Analysis

The search process retrieved 42 relevant studies. They were written by 111 authors based on institutions from 23 different countries, distributed in four continents. Table 5 shows the number of articles per country, which was derived from the address of the first author of the articles. The country with the most publications was the United States of America (USA) (*n* = 18), followed by Brazil and the Netherlands (*n* = 2).

**TABLE 5:** Number of articles per country*.*

Country	Number of articles
United States	18
Netherlands, Brazil	2
Austria, Bulgaria, China, Estonia, Germany, Greece, India, Indonesia, Italy, Jordan, Pakistan, Romania, Singapore, South Africa, Spain, Taiwan, Turkey, United Kingdom, Uruguay and Spain	1
**Total**	**42**

**TABLE 6:** Evidences that Learning Analytics improves high school student performance.

Evidence	Number of articles (%)
No evidence	25 (59.52%)
Positive with empirical evaluation	8 (19.05%)
Positive without empirical evaluation	9 (21.43%)
Negative with empirical evaluation	0 (0%)
Negative without empirical evaluation	0 (0%)
**Total**	**42 (100%)**

**FIGURE 2:**
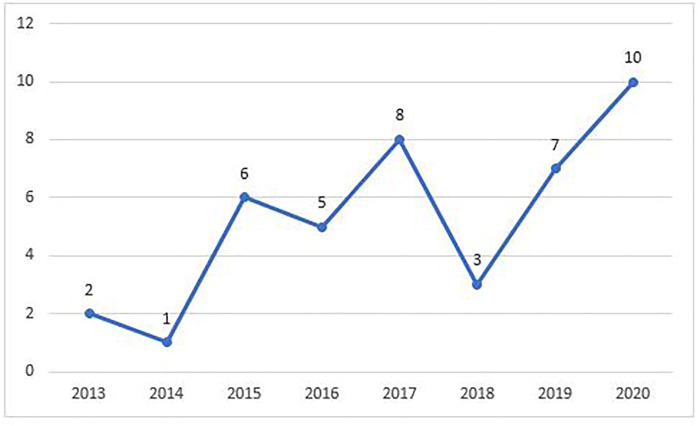
Distribution of the papers included in the review across years.

Figure 2 Shows the distribution of studies per year of publication. The figure shows an increase in publications on learning analytics for high schools in recent years. In the last 4 years (2017–2020), there were two times more articles than in the early years (2010–2016). During the period analyzed, the years with the fewest publications were 2010, 2011 and 2012 (*n* = 0) and the years with most publications were 2020 (*n* = 10).

Finally, the most common keywords used in the selected articles with their respective frequency were: students (16), Learning Analytics (12), data mining (12), teaching (6), high school 5) education (5), high school students (5), computer-aided instruction (5), E-learning (5), learning systems (4), learning (4), curricula (4), feedback (4), machine learning (4), and data visualization (4). The top five keywords–students, learning analytics, data mining, teaching and high school–reflect precisely the research theme of this SLR.

### 5.2 Results of Research Questions

#### 5.2.1 RQ1: What are the Approaches for the use of Learning Analytics in High Schools?

The first research question raised in this SLR was related to the main educational goals of using learning analytics in the context of high school. Table 7 shows that analyses of students’ learning outcomes and students’ learning processes were the most important goals in this context. It is also relevant that among the papers included in the SLR, more than 10% focused on teacher support.

**TABLE 7:** Main educational goals in using learning analytics in high schools*.*

Goal	Number of articles (%)
Predict and enhance students learning outcomes	18 (42.85%)
Analyze students’ learning processes	11 (26.19%)
Support teachers’ decisions and reflection	5 (11.91%)
Support writing activities	3 (7.14%)
Others	5 (11.91%)
**Total**	**42 (100%)**

The main goal of applying learning analytics in high school was to predict student droput or to predict and enhance students’ learning outcomes. There was an effort to predict students’ grades in order to provide support and personalization (Blasi, 2017). For instance, Wandera et al. (2019) proposed several models to predict school pass rate in order to support higher-level decision making. Similarly, Aguiar et al. (2015) developed performance prediction models to help schools more efficiently allocate limited resources by prioritizing students who are most in need of help and target intervention programs to match those students’ particular needs.

In addition to the prediction of final grades, learning analytics in high school was used to predict school dropout (Lakkaraju et al., 2015; Filho and Adeodato, 2019; Baker et al., 2020), discovering clues to avoid middle school failure at early stages (Jiménez-Gómez et al., 2015), and to assist the education department or policymakers to predict the number of graduating and dropout students (Yousafzai et al., 2020).

The second most cited educational goal identified was to analyze students’ learning processes. Most works aligned to this goal investigated students’ participation in assessment and educational games using log data. For instance, multiple data sources, including self-report models and activity logs, were collected from 25 classes at a senior high school in northern Taiwan, aiming at the application of supervised and unsupervised lag sequential analysis (LSA) for examining students’ learning processes (Wen et al., 2018). Grover et al. (2017) and Manske and Hoppe (2016) also used log data to evaluate students’ participation in computational thinking activities and the Go-Lab portal, respectively. The main goal of both studies was to support students in reflecting on personal knowledge building by visualizing their log data. Another application in the same direction was the analysis of the behavior of solo and collaborative groups of students engaged with educational games to evaluate differences between students interactions in these two profiles based on in-game log data, which is a novel approach that scales up to large groups (Ruipérez-Valiente and Kim, 2020).

The support of teachers’ decision making and reflection was also found relevant in the papers retrieved. In this context, Chen (2020) proposed an approach to explaining how teachers’ behavior influences classroom teaching performance. In this direction, different papers proposed data visualization tools, real-time learning analytics (Berland et al., 2015), and computer-based assessment data visualization (Admiraal et al., 2020) to assist teachers’ decision making.

Another relevant topic found within the scope of this research question was the support of writing activities (Palermo and Wilson, 2020). The most complete paper on this topic is the description of the iStart tool, which provides formative feedback in written assessments (Allen et al., 2017). This study suggested that dynamic visualizations and analyses can be used as a step towards more adaptive educational technologies for literacy and any system that collects students’ natural language responses. This approach provides a strong initial foundation because it demonstrates the feasibility of such measures for modeling student performance (Allen et al., 2017).

Finally, we also found papers related to real-time adjustable feedback (Lee et al., 2019), analyses and classification of students’ sentiments towards the educational process (Marcu and Danubianu, 2020), and direct mapping between learning traces typically gathered for learning analytics and a theoretically grounded model of cognition (Seitlinger et al., 2020).

#### 5.2.2 RQ2: What are the Approaches for the use of Learning Analytics in High Schools?

Table 8 shows the approaches used in the adoption of learning analytics for high schools. The majority of the applications are related to visualizations (in the distillation of data for human judgment category), prediction and relationship mining. The other two categories (discovery with models and clustering) were covered in less than 10% of the papers each.

**TABLE 8:** Main data analysis approaches used studies on learning analytics in high schools.

Approach	Number of articles (%)
Distillation of data for human judgment	15 (35.71%)
Prediction	11 (26.19%)
Relationship Mining	9 (21.43%)
Discovery with models	4 (9.53%)
Clustering	3 (7.14%)
**Total**	**42 (100%)**

In learning analytics, visualization is one of the main topics of research and practice. In the categories that we used for evaluating the approaches used in high schools, visualizations are categorized as the distillation of data for human judgment. There were several examples of the application of visualizations to support different stakeholders in high schools. For instance, Chen (2020) proposed the Visual Learning Analytics (VLA) approach combining the perspectives of learning analytics and visual analytics to understand education. The approach was applied to give support to a video-based teacher professional development program. More specifically, this study compared how conventional knowledge-based workshops and the VLA-supported hands-on workshops influenced teacher beliefs about the usefulness of classroom talk (based on the Academically Productive Talk approach), self-efficacy in guiding classroom talk, and actual enactment of dialogic teaching in the classroom (Chen, 2020). Results showed that VLA-supported teacher professional development was an effective approach to improving teachers’ methodology and development of dialogic teaching (Chen, 2020).

Visualizations were also employed to provide reflections for high school language teachers (Admiraal et al., 2020). In this study, the authors used data collected from a computer-based environment called Got it Language^2^ to provide insights into how teachers’ classroom instruction was perceived by students (Admiraal et al., 2020). The proposed dashboard supported teachers in adapting their lesson plans and instructions to improve students’ performance (Admiraal et al., 2020). Similarly, Papamitsiou and Economides (2015) proposed the use of temporal Learning Analytics visualizations for increasing student awareness during assessment. Visual representations of student-generated log data during learning activities support students and instructors in interpreting them intuitively and perceiving hidden aspects of these data quickly. Finally, the learning analytics dashboards were also used to support the feedback process (Lee et al., 2019), provide information about the use of a virtual learning environment (d’Anjou et al., 2019), and promote collaborative knowledge construction (Manske and Hoppe, 2016).

There were two main lines of work in terms of prediction: predicting students at risk and student learning outcomes. This finding is closely related to the results of RQ1, but it includes a broader view of prediction in the context of high schools. For example, Baker et al. (2020) analyzed a set of complex patterns and factors like student attendance, grades (and their changes), course-taking, and disciplinary records, using a logistic regression algorithm to predict dropout of high school students (Baker et al., 2020). The model predicting dropout achieved an area under the ROC curve (AUC) of 0.76 and the authors identified that the total number of non-correctible dress code violations, the number of in-school suspensions and the standard deviation of grades in the current semester were the most predictive features. Using a similar approach, Aguiar et al. (2015) used random forest and logistic regression models for early prediction of students at risk of not graduating from high school. The authors suggested that these predictions can be used to inform targeted interventions for these students, hopefully leading to better outcomes (Aguiar et al., 2015).

In terms of prediction learning outcomes, Yousafzai et al. (2020) used supervised machine learning techniques with students’ demographics information and results of previous exams to predict the students’ overall performance in Pakistan. The classifiers reached an accuracy higher than 95%. In the same direction, Wandera et al. (2019) and Blasi (2017) applied deep neural network architectures to predict the final grades of high school students. Both papers reached accuracy of approximately 90%. However, Wandera et al. (2019) also included the SHAP (SHapley Additive exPlanations) analysis (Lundberg and Lee, 2017) to provide insights into the most relevant features for the problem.

Relationship mining aims at analyzing association, sequential, and collaborative patterns in educational data. In this context, learning analytics was used to examine student practices in different learning scenarios. For instance, Ruipérez-Valiente and Kim (2020) proposed a system to investigate the influence of gameplay style (solo or collaborative gameplay) of students using the Shadowspect platform. The authors evaluated the performance of the students using engagement metrics and graph analysis. In another context, learning analytics was used to measure the acquisition of computational thinking in block programming environments in high school curricula over time (Grover et al., 2017). The main goal of the study proposed by Grover et al. (2017) was the proposal of a framework that formalizes a process with a hypothesis-driven approach using evidence-centered design. Relationship mining was also employed to evaluate collaboration in real-time among novice middle school, using graphical analysis based on log data (Berland et al., 2015), and to understand students’ partners in the use of EcoSurvey, a collaborative tool for Biology classes (Quigley et al., 2017).

The papers related to discovery with model categories focused on the use of previous models in new contexts. For instance, Palermo and Wilson (2020) adopted an automated writing evaluation system, called MI Write, in schools in North Carolina, United States. This system supports the provision of argumentative writing prompts for students in real-time. In another context, pre-trained machine learning models were also used to predict students at-risk in new school districts (Coleman et al., 2019) and build learning analytics visualizations for Bulgarian school education (Gaftandzhieva et al., 2020).

Finally, the category with the fewest papers was clustering. In this case, data analysis techniques were applied to assess the student-student and student-teacher interaction to see how the information extracted from clustering analysis can affect teaching strategies, especially those related to strategic group formation and school management (Ponciano et al., 2020). Lailiyah et al. (2019) benefited from clustering algorithms to identify student behavior and preferences in a high school context. The authors collected data from questionnaires and used traditional clustering algorithms (k-Means and Fuzzy C-Means) to aggregate students with similar characteristics.

#### 5.2.3 RQ3: Which machine Learning Techniques Have Been Used to Support Learning Analytic Systems in High Schools?

Table 9 presents the list of the most used machine learning techniques in the retrieved articles. It shows a preference for traditional algorithms in comparison to deep neural networks. Moreover, the white box nature of decision trees algorithms could explain why they were at the top of the list. It is important to mention that a few papers used more than one algorithm (it justifies why the total sum in Table 8 is larger than 42) and others did not provide enough information about the algorithms.

**TABLE 9:** Main machine learning techniques applied to the context of high school*.*

Technique	Number of articles
Decision Trees (DT)	12 (21.05%)
Probabilistic classifiers	8 (14.03%)
Logistic regression	7 (12.28%)
Natural Language Processing	6 (10.52%)
Artificial Neural Network (ANN)	5 (8.77%)
Clustering	4 (7.01%)
No details	15 (26.34%)

The techniques listed in this section are highly related to the educational goals presented in section 5.2.1. The papers related to predicting and enhancing students learning outcomes in general adopted traditional machine learning algorithms such as decision trees, naïve Bayes, support vector machines, logistic regression, and neural networks (Aguiar et al., 2015; Lakkaraju et al., 2015; Blasi, 2017).

The papers related to analyzing students’ learning processes predominantly applied decision tree algorithms and clustering techniques (Grover et al., 2017; Wen et al., 2018). It is important to highlight that traditional decision tree and random forest algorithms were used in seven and three papers, respectively. Only two papers used the state of the art decision tree algorithms (Adaboost and XGBoost) (Jiménez-Gómez et al., 2015; Lakkaraju et al., 2015). Moreover, the k-means algorithm was used in 75% of the papers related to clustering analysis (Abadi et al., 2018; Lailiyah et al., 2019).

As expected, natural language processing (NLP) techniques were found in papers that support writing activities. For instance, Allen et al. (2017) used different language models and resources, based on the iSTART system, to analyze the dynamics of discourse in a reading strategy, and Palermo and Wilson (2020) proposed the use of MI Write system to evaluate written activities in different contexts.

#### 5.2.4 RQ4: What evidence, if any, Shows That Learning Analytics Improves the Performance of Students in High Schools?

This section reports the results about the evidence found on the impact of learning analytics in the context of high school Table 6. By evidence, we mean scientific indication that (Ferguson and Clow, 2017): learning analytics improves learning outcomes, learning analytics improves learning support and teaching, and learning analytics is taken up and widely used, including deployment at scale.

Unfortunately, the majority of the papers retrieved (25–59.52%) did not present any details that could ensure this kind of evidence. Moreover, none of them reported negative evidence. We divided the papers with positive evidence with and without empirical evaluation, which related to the papers that reported the adoption of learning analytics in practice and the papers reporting only experimentation, respectively.

The papers that reported empirical evaluation presented the practical use of learning analytic tools by students or teachers. For instance, Admiraal et al. (2020) reported that learning analytics improved language learning. In this case, the teachers’ perceptions were: 1) the low-performing students were triggered to act based on the automatic feedback received; 2) the computer-based test enhanced the learning opportunities as students practiced their language skills. Another approach was the analysis of models created in previous courses to a new cohort of students. For instance, Berland et al. (2015) demonstrated the positive effect of learning analytics for the formation of groups in the context of pair programming activities. This approach improved students’ performance, enabling them to develop better and more efficient programs. Similarly, Palermo and Wilson (2020) presented the improvement of students’ writing quality after the adoption of an automated writing evaluation.

#### 5.2.5 RQ5: What are the Challenges in Using Learning Analytics in High Schools?

The majority of the papers retrieved in this SLR did not explicitly highlight any challenges of the application of learning analytics in high schools. However, the main issues raised were related to data quality, especially when considering different sources of data (Filho and Adeodato, 2019; Yousafzai et al., 2020), and privacy concerns (d’Anjou et al., 2019; Jadav et al., 2017).

Other technical issues related to possible data collections were also considered. For instance, problems related to internet connectivity (Berland et al., 2015) and the number of devices per classroom (Monroy et al., 2013) are the main concerns in this direction. The other limitations stated by the selected papers are related to the specificity of each study.

## 6 Discussion

This section presents the main insights of this systematic literature review, in the light of previous literature on learning analytics. Moreover, we highlight the main aspects that should be addressed by studies that adopt Learning Analytics in the high schools’ context.

The first research question analyzed in this study focused on the main educational goals for using learning analytics in schools. We found that these goals are focused on predictions of learning outcomes rather than supporting instructors and students in the decision-making process, or understanding students’ behavior. This is a characteristic of the initial articles in the field of learning analytics (Charitopoulos et al., 2020; Joksimović et al., 2019). Recent literature on learning analytics proposes the application related to a wide variety of goals that are not focused on prediction, such as providing feedback (Tsai et al., 2021b; Pardo et al., 2019), supporting counseling sessions (De Laet et al., 2020), analyzing students’ tactics and strategies (Matcha et al., 2019c,a), and understanding students’ knowledge construction in online discussions (Rolim et al., 2019; Ferreira et al., 2021; Neto et al., 2021).

Our review did not reveal papers suggesting the analysis of school context, which is considered a critical activity to ensure successful learning analytics adoption (Gasevic et al., 2019; Falcão et al., 2020; Tsai et al., 2021a). In general, the papers retrieved in this review did not include the stakeholders in the process of creating analytic models, tools, and systems, even the ones focusing on supporting teachers (Michailidis et al., 2017). The lack of understanding of the school context and the focus on prediction reinforce that the adoption of learning analytics for high schools is still taking its first steps.

Concerning the second research question, about the main technical approaches used in learning analytics for high schools, it is possible to draw a direct comparison of learning analytics for high schools and higher education. Viberg et al. (2018) found that in the context of higher education, predictive methods (including regression and classification) were the most frequent (32%), followed by relationship mining (24%) and distillation of data for human judgment (24%). In our analysis, the most important approach found was the distillation of data for human judgment, with 35.71%, followed by predictions (26.19%) and relationship mining (21.43%).

Although the top-3 categories coincide between the findings of the current review and the Viberg et al. (2018) review, the discrepancy between the number of papers related to predictive methods for higher education and the distillation of data for human judgment for high school is relevant. Two main factors may explain this result: 1) predictive methods applied in higher education, in general, are centered on the identification of aspects related to learning processes (Viberg et al., 2018), while the majority of the models for high schools focuses on learning outcomes (Jiménez-Gómez et al., 2015; Blasi, 2017); 2) the dashboards proposed by papers related to high schools (d’Anjou et al., 2019; Chen, 2020) are relatively simpler in comparison with those related to higher education (Matcha et al., 2019b). Based on these findings, it is possible to recommend that researchers developing LA for high schools should include more analysis related to the learning process to improve the quality of the dashboards to support students’ and teachers’ decision-making.

In terms of machine learning algorithms used in the studies included in the current review (third research question), almost 50% benefited from white-box algorithms such as decision trees and logistic regression. The use of white-box algorithms is a trend, and even a recommendation, in educational applications of machine learning (Almohammadi et al., 2017; Nistor and Hernández-Garcíac, 2018; Charitopoulos et al., 2020). Previous literature reviews in the context of higher education (Leitner et al., 2017) and workplace learning (Ruiz-Calleja et al., 2017, 2021) also indicate the trend of using different decision trees and regression algorithms to create models and perform analysis in the educational data. For instance, learning analytics methods rely on the fact that there is a person involved in the decision loop (Siemens and Gasevic, 2012), and white-box models provide more information about the decision made by the classifier.

The fourth research question revealed the lack of evidence on the success of the application of learning analytics reported in the studies included in the current review, suggesting that learning analytics is in the early days of adoption in high schools. The majority of the papers did not indicate any evidence demonstrating that learning analytics could be efficient when applied to practice. On the other hand, in the context of higher education, learning analytic applications are starting to scale to an institutional level (Viberg et al., 2018; Herodotou et al., 2020).

Another reflection of limited maturity in adopting learning analytics in high schools is the reduced number of challenges reported in the studies included in the current SLR (fifth research question). The central claims in this direction were related to data quality, privacy, and technical issues (e.g., internet connectivity and number of devices per classroom). While in the case of higher education institutions, the literature reported more complex concerns, such as stakeholders’ involvement, understanding of institutional needs, and more general ethical, privacy protection, and data governance issues (Gasevic et al., 2019; Cechinel et al., 2020).

Based on the results described in this paper, we highlight the following aspects to be considered by researchers working with learning analytics in high schools:

1. **Institutional diagnosis**: A very important issue we have noticed in the papers included in the SLR is the absence of a methodological process to understand schools needs and context for learning analytics adoption. In this direction, several frameworks have been proposed for adoption of learning analytics in higher education. For instance, Tsai et al. (2018) proposed SHEILA, a framework that guides higher education institutions in adoption of learning analytics by providing relevant instruments for involvement of relevant stakeholders and by documenting actions taken, policy questions, and challenges commonly experienced by institutions. SHEILA has widely been used across the world (Maldonado-Mahauad et al., 2018; Broos et al., 2020; Falcão et al., 2020; Vigentini et al., 2020)). Therefore, SHEILA or similar frameworks could be used as a good starting point to understand the needs of high schools regarding learning analytics.
2. **Ethical concerns**: In a similar direction, it is important to consider ethical issues (Pardo and Siemens, 2014). For instance, in the case of students under 18, who is responsible for the duty of care of the data? Which kind of data should be analyzed? Would it be ethical for the schools to provide mobile and wearable devices for students?
3. **Learning analytic techniques**: learning analytic applications, in general, focus on the learning process and not only on the learning outcome (Joksimović et al., 2019). For instance, in the case of using learning analytics to promote feedback on process level (Hattie and Timperley, 2007), it is necessary to be able to identify learning processes from data available in schools, and not only the outcome. Therefore, studies in high schools should adopt techniques such as social network analysis (Knoke and Yang, 2019), epistemic network analysis (Shaffer et al., 2009), and process mining (Van Der Aalst, 2012) instead of just using machine learning algorithms.
4. **Explainable artificial intelligence**: Although this literature review highlighted the importance of using white-box machine learning methods, recent literature proposes the combination of deep learning with explainable artificial intelligence techniques in the analysis of educational data (Kovalerchuk et al., 2021). Specifically in learning analytics, explainable artificial intelligence is still at an initial adoption step, but researchers have already reported positive results (Verbert et al., 2020; Ochoa and Wise, 2021).

## 7 Limitations

The primary limitation of this study is related to the search process in which we only focused on papers that contain the specific keyword “high school”. This could potentially exclude papers that describe the adoption of learning analytics in secondary schools, the terminology used by other countries to refer to high school in their educational systems.

Secondly, a few papers had limited information about the methods and techniques used, which led to several coding some studies with labels such as “no details” and “no evidence” in some of the categories that were analyzed in the current review. We decided to keep these papers nevertheless because they contained relevant information to at least one research question. However, it is important to highlight that we performed a critical assessment to assure the quality of the paper.

Finally, this review did not focus on papers in the fields that are related to learning analytics such as educational data mining and artificial intelligence in education, which could broaden the reach of this SLR.

## Data Availability

The raw data supporting the conclusions of this article will be made available by the authors, without undue reservation.

## Footnotes

1
https://www.solaresearch.org

2
https://www.thiememeulenhoff.nl/got-it
